# Noninvasive measurements of tissue perfusion in critical limb ischemia

**DOI:** 10.1007/s00772-018-0368-x

**Published:** 2018-03-02

**Authors:** U. Rother, W. Lang

**Affiliations:** Department of Vascular Surgery, Erlangen University Hospital, Krankenhausstraße 12, 91056 Erlangen, Germany

**Keywords:** Critical limb ischemia, tcpO_2_, O2C, MSOT, Fluorescence angiography, Kritische Extremitätenischämie, tcpO_2_, O2C, MSOT, Fluoreszenzangiographie

## Abstract

Critical limb ischemia (CLI) remains a challenge for an interdisciplinary therapeutic team due to chronic nonhealing wounds. Against this background, there is a necessity of quality control after revascularization. Beside the isolated evaluation of the macrocirculation by Ankle-Brachial or Toe-Brachial Index measurements, the microcirculation as an additional important factor of wound healing often remains underestimated. The following article gives an overview about the current investigation methods for noninvasive perfusion control of the CLI patient. Therefore, transcutaneous oxygen pressure (tcpO_2_), the “oxygen-to-see” method which is a combination of white light tissue spectrometry and laser-Doppler flowmetry, fluorescence angiography with indocyanine green, and multispectral optoacoustic tomography will be described.

## Zusammenfassung

Die kritische Extremitätenischämie stellt aufgrund nicht heilender Wunden nach wie vor eine Herausforderung an ein interdisziplinär behandelndes Team dar. Vor diesem Hintergrund ist es notwendig, suffizient Erfolgskontrollen einer Revaskularisation durchführen zu können. Neben der Beurteilung der Makrozirkulation in Form von Knöchel-Arm- und Zehen-Arm-Index-Messungen wird häufig die Relevanz der Mikrozirkulation unterschätzt. Um allerdings ein Gesamtbild der Durchblutungssituation zu erhalten, sollte diese nicht außer Acht gelassen werden. Dargestellt werden sollen daher aktuelle Methoden der nicht invasiven Perfusionsanalyse. Hierzu zählen die transkutane Sauerstoffpartialdruckmessung, das „Oxygen-to-see“-Verfahren – eine Kombination aus Weißlichtspektrometrie und Laserdopplerflowmetrie – sowie die Fluoreszenzangiographie durch Indocyaningrün. Ein Ausblick auf mögliche zukünftige Verfahren wie die multispektrale optoakustische Tomographie wird ebenso gegeben.

## Introduction

Despite advances in treatment methods, chronic critical limb ischemia (CLI) remains a challenge for the interdisciplinary team. Non-healing, chronic wounds are particularly relevant in this context. According to the current literature, chronic wounds fail to heal despite successful macroscopic revascularization in 10–18% of cases [1–3]. Given these figures and in addition to considering the macrocirculation in isolation, it is essential to obtain an overall impression of perfusion status, which also includes an assessment of microcirculation. Therefore, this article describes noninvasive methods of perfusion measurement.

## Transcutaneous measurement of oxygen partial pressure

Transcutaneous measurement of oxygen partial pressure (tcpO2) is a noninvasive, polarographic method to measure pO2 at the skin surface. It can also be used indirectly to draw conclusions about systemic arterial oxygen pressure. As part of the measurement process, heated probes (Clark-type measuring electrodes) are applied to the skin. Oxygen molecules that diffuse through the skin to the surface are reduced between the electrodes of the probe to which voltage is applied. The reduction current produced in this way is proportional to the reduced oxygen molecules, enabling conclusions to be drawn about pO2 [4]. The attached probes can be heated to different temperatures, thereby producing different situations. If the probes are heated to 37 °C, blood vessel reactivity is to a large extent maintained, thereby permitting functional tests to be performed. The majority of available devices use a temperature between 43–45 °C, which measures skin perfusion at maximum hyperemia [4]. An advantage of the tcpO2 measurement technique is that there are standardized values for vascularly healthy individuals. For example, Gothgen and Jacobsen showed an average partial oxygen pressure of 50 mm Hg on the dorsum of the foot in vascularly healthy patients [5]. Therefore, an attempt was made to assign individual mean pO2 to different stages of arterial occlusive disease (AOD); however, it could only be shown that symptomatic stages of AOD (Fontaine stages II–IV) are associated with significantly reduced pO2 values; values of 0–10 mm Hg were defined as hypoxic [5]. The tcpO2 measurements are also used to determine amputation levels. Thus, repeated preoperative measurements of tcpO2 values should include at least one pO2 over 5 mm Hg; lower values are associated with an increased likelihood of impaired wound healing and reamputation. Reliable wound healing can be expected at values over 30 mm Hg [5]. A limitation of this method is its relatively long measuring time. A heating phase of approximately 20 min is first required in order for a measurement to be taken. The resting tcpO2 level is defined as the level that changes less than 2 mm Hg in the 2 min following a 10-min response time [6]. In addition, numerous influencing factors that significantly limit the reproducibility and inter-individual comparability of this measurement method have been shown, including ambient temperature, skin changes such as inflammation and necrosis, the temperature to which the electrode is heated, age, smoking, and gender [4].

## Oxygen-to-see (O2C) method

**Fig. 1:**
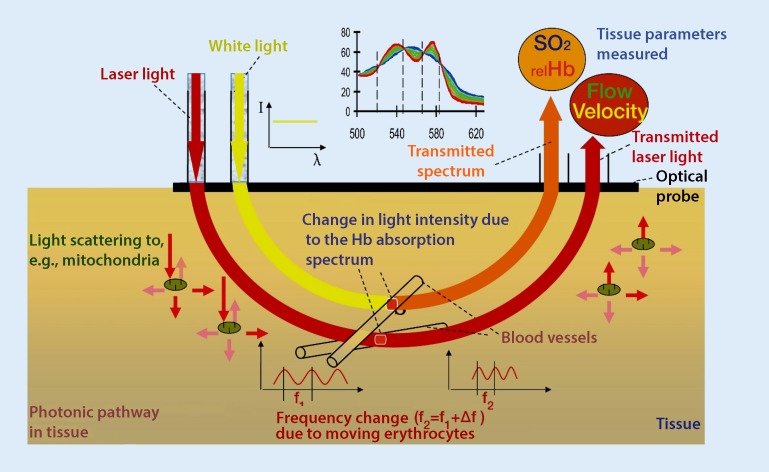
The O2C measuring principle: a combination of white light spectrometry and laser Doppler flowmetry (courtesy of LEA Medizintechnik GmbH)

The O2C (LEA Medizintechnik GmbH, Gießen, Germany) method is an optical measuring technique that combines white light spectrometry and laser Doppler flowmetry (see Fig. 1). This technique enables three parameters to be mapped: oxygen saturation (sO2), relative hemoglobin (rHb) and blood flow (flow). This measurement method irradiates tissue with a broad band light source as well as light from a laser source. The sensor measures the light re-emitted by the tissue. The white light source spectrum in the wavelength range of 500–850 nm is used to determine sO2 and rHb. A laser light source with a wavelength of 830 nm is used to determine blood flow. Blood flow is provoked by a Doppler shift in the emitted laser light through the erythrocytes moving on the microcirculatory level, and blood flow is derived in relation to the total erythrocyte count [7–9]. Oxygen saturation of hemoglobin is determined by means of the white light source and the absorption of irradiated blood. The absorption of the emitted white light yields the rHb. The penetration depth of this measurement technique depends on the selected probe and its respective separation, whereby penetration depths of up to 8 mm are possible. In particular the parameters sO2 and blood flow have proved to be relevant in the diagnosis of AOD, whereby patients should be examined both in a resting and a provocation position [8]. Under provocation, the foot is raised to a height of 68 cm, corresponding to a perfusion pressure of approximately 50 mm Hg. Below this, both sO2 levels and flow measurements should not fall below 10 arbitrary units (flow) or 10% (sO2), which would otherwise define the stage of CLI [10, 11]. Absolute values are also available for the determination of amputation levels, whereby a cure can be expected here if, after repeated measurements, mean resting sO2 levels are 30% and no values should fall below 15% [12]. This technique is also used for periprocedural perfusion measurements in the foot following balloon angioplasty. Measuring perfusion in the foot angiosome following isolated crural measurement showed that there was no significant difference between direct and indirect revascularization on the microcirculatory level: on the contrary, an improvement in overall foot perfusion was seen following revascularization [13, 14].

## Fluorescence imaging techniques

Fluorescence is an optical phenomenon in which substances absorb light by means of electromagnetic radiation and thereby become excited. Light that can be visualized is then emitted from the substance. Fluorescence imaging techniques can be divided into those that can be visualized using internal contrast and those that require a contrast agent [15]. The autofluorescence technique takes advantage of the property of tissue to self-fluoresce following light irradiation at a certain wavelength [16]. This technique is primarily used in endoscopy. For example, early-stage bronchial carcinoma can be differentiated from healthy lung tissue, as well as healthy from diseased intestinal mucous membranes [16]. Other fluorescence imaging techniques used in medicine generally require a fluorochrome to increase contrast. The substances currently used for this include fluorescein and indocyanine green (ICG), which can be administered intravenously [17, 18]. They are used to visualize tissue perfusion. In addition, 5‑aminolevulinic acid (5-ALA) is used in neurosurgery to visualize brain tumors [19]. Increased uptake of 5‑ALA occurs in glioblastoma cells, for example, which convert the dye into protoporphyrin IX, a fluorescent molecule. This enables a differentiation between pathological and healthy tissue.

Fluorescence angiography using ICG is currently finding increasing application in vascular surgery; therefore, ICG is described in more detail below.

## Fluorescence angiography with indocyanine green

**Fig. 2:**
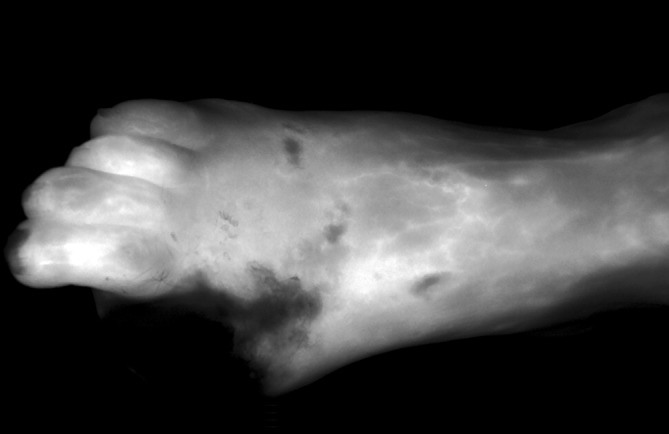
Fluorescence angiography with indocyanine green (SPY system, NOVADAQ, STRYKER, Kalamazoo, USA) following hallux amputation

**Fig. 3:**
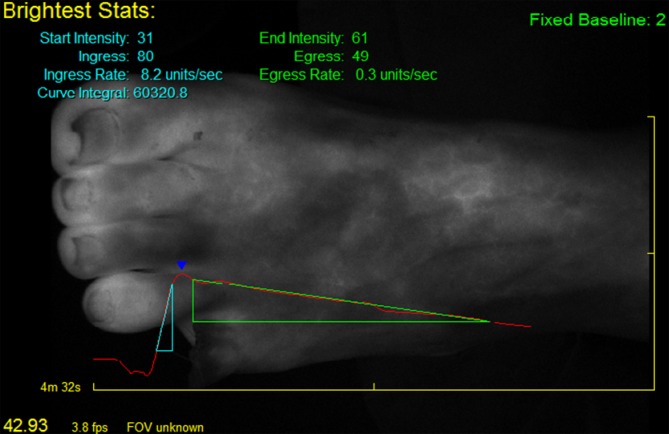
Quantification of fluorescence angiography with indocyanine green (SPY-Q, NOVADAQ). Fluorescence intensity is quantified using a 256 gray scale, thereby enabling inflow and outflow to be assessed

Fluorescence angiography with ICG is a technique to visualize tissue perfusion. The ICG is administered intravenously and the target organ irradiated using a near-infrared light source, which excites the dye to fluorescence and makes tissue perfusion visible via a camera system (see Fig. 2). The wavelengths of ICG fluorescence angiography are outside the visible range, whereby emission wavelengths are 805 nm and the absorption spectrum 830 nm. Following intravenous administration, the ICG primarily binds to plasma proteins and elimination takes place via the hepatobiliary system [20]. A water-soluble tricarbocyanine dye, ICG has been used in medicine for some considerable time. It was first used in 1959 to assess liver function and measure cardiac output [20–22]. Since 1975, fluorescence angiography has been used primarily in ophthalmology, where it enables an assessment of blood flow in the retina and choroid [23]. Particularly in recent years, fluorescence angiography has also found increasing application in reconstructive plastic surgery where it enables flap perfusion borders to be determined and perforator vessels identified [24, 25]. Fluorescence angiography has also become of increasing interest in vascular surgery, since it enables the visualization of perfusion of the entire foot. It is also possible to quantify this imaging method, since it is performed in gray-scale mode, thereby enabling individual fluorescence intensities to be categorized into individual gray scales. As part of this, it is possible to assess the washout dynamics of ICG in the periphery (see Fig. 3).

Fluorescence angiography visualizes the perfusion of the entire foot

A number of studies have shown a correlation between measurements of the ankle-brachial index, the toe-brachial index, TcpO2 measurements, and perfusion data from fluorescence angiography [26–28]. Use of the technique becomes problematic in patients with peripheral arterial occlusive disease (PAOD) Fontaine stage IV and concomitant foot infection. In such cases, imaging tends to overstate perfusion in the infected areas, thereby precluding adequate evaluation [26]. Approaches investigated in recent studies involving attempts to use this perfusion method to evaluate amputation limits and predict the further healing process show promise. Also, in visceral surgery the procedure is increasingly gaining in popularity, since it can be used to determine bowel resection lines in mesenteric ischemia and perfusion in anastomosed bowel regions [29, 30].

## Multispectral optoacoustic tomography

**Fig. 4:**
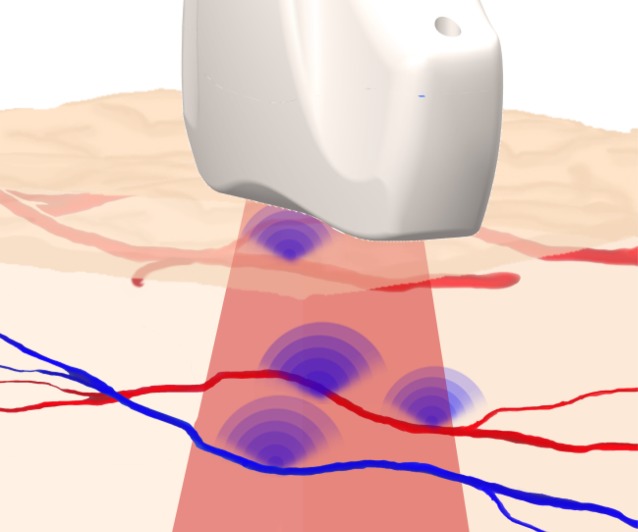
The principle of multispectral optoacoustic tomography (MSOT): tissue is illuminated with nanosecond laser pulses. Absorption of light energy by absorbers in the tissue causes a slight rise in temperature and thermoelastic expansion. The ultrasound waves thus generated are registered with a hand-held detector (courtesy of iThera Medical GmbH)

**Fig. 5:**
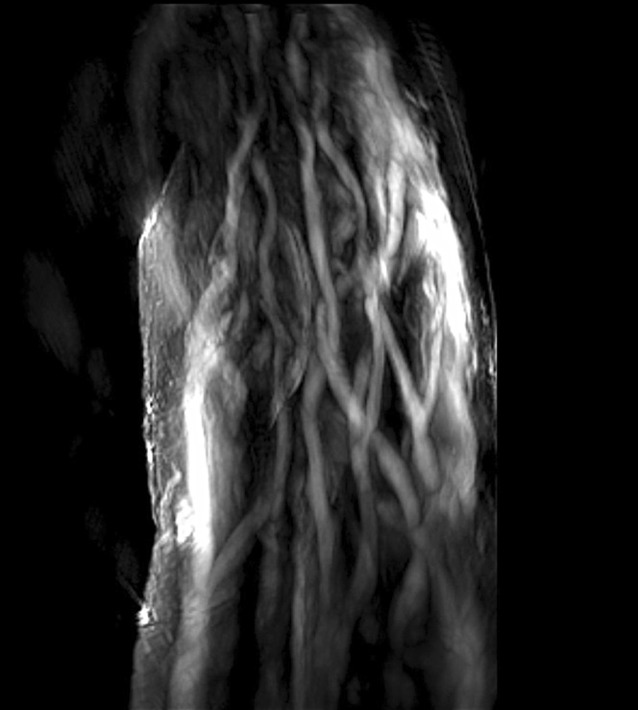
Multispectral optoacoustic tomography (MSOT) image of the vascular plexus in a test subject, taken with a 270° detector (MSOT inVision; courtesy of iThera Medical GmbH)

Multispectral optoacoustic tomography (MSOT, iThera Medical GmbH, Munich, Germany) is a novel and promising technique of noninvasive perfusion measurement, which may yield new insights into microcirculation in the future. The technique combines optical imaging with conventional ultrasound. The target tissue is illuminated with pulsed laser light in the near infrared range. The laser pulses are absorbed by the tissue, where they cause an extremely slight and transient rise in temperature (see Fig. 4). This results in thermoelastic expansion, which generates an ultrasonic wave that can be visualized using highly sensitive detectors. Light excitation at different wavelengths enables conclusions to be drawn on tissue composition from the absorption spectrum. Thus, the procedure can render intrinsic absorbers visible (e. g., oxygenated hemoglobin, deoxygenated hemoglobin, melanin, lipids); however, it can also be used with contrast agents [31, 32]. For instance, ICG or methylene blue can be used. A study recently published by Taruttis et al. demonstrated for the first time, the benefits conferred by this technique in the visualization of blood vessels [33]. The major foot arteries (dorsal pedal artery, posterior tibial artery), as well as tiny arterioles of the foot microcirculation up to a diameter of 100 µm, could be visualized in healthy subjects. It was also possible to visualize the venous plexus. Oxygenated and deoxygenated hemoglobin were used as absorbers (see Fig. 5). Due to the ability to simultaneously measure and spectrally separate oxygenated and deoxygenated hemoglobin, one is able to not only visualize the microcirculation, but also draw conclusions on the degree of oxygenation [33]. The additional use of a contrast agent such as ICG could be very promising in the future, since it might also permit statements to be made about perfusion dynamics. Furthermore, MSOT can also be used to visualize perfusion, i. e., hemoglobin content and overall tissue oxygenation, e. g., in the intestinal wall in patients with chronic inflammatory bowel diseases [34]. At present, the MSOT technique is only available in the context of studies, since it does not yet have valid CE certification.

## Summary

In view of the reporting standards of chronic lower extremity peripheral artery disease recently published by the Society of Vascular Surgery and the multitude of options to analyze microcirculatory perfusion, any examination of microcirculation in patients with CLI should be accompanied by a determination of the macrocirculation using the ankle-brachial index and duplex sonography in order to obtain an overall picture of perfusion status [35]. Only then is it possible to adequately classify the perfusion status of the foot and categorize a wound according to the wound ischemia and foot infection (WIfI) system [36]. The measurement of tcpO2 and the O2C method are without doubt the standardized methods, since reference values are available here, whereby tcpO2 measurement requires more time and is subject to a number of influencing factors. Both fluorescence angiography and the MSOT technique offer the additional option to visualize perfusion dynamics and represent promising techniques for the future.

## Conclusion

- In order to fully assess the perfusion status of the foot in CLI, both microcirculation and macrocirculation need to be evaluated.
- Standard values that define a critical perfusion status on the microcirculatory level are available for tcpO2 and O2C measurement (tcpO2: <10 mm Hg, O2C: sO2 <10%).
- Although fluorescence angiography offers the option to visualize the microcirculation, standardized norm values are still lacking.
- The MSOT represents a technique for the noninvasive visualization of microcirculation that holds promise for the future; however, more studies are needed before it can be implemented in a standardized manner.
